# Respiratory trajectory and risk of death or moderate-to-severe bronchopulmonary dysplasia in very preterm infants: a nine-year cohort study

**DOI:** 10.3389/fped.2026.1843270

**Published:** 2026-06-01

**Authors:** Talal Aljarbou, Abdulaziz Homedi, Saad Alshareedah, Maather Almutairi, Moudhi Alhumaidi, Nouf Alayyar, Lara Almobiedh, Mohamed Sufyani, Saif Alsaif, Kamal Ali

**Affiliations:** 1 Neonatal Intensive Care Department, King Abdulaziz Medical City, Riyadh, Saudi Arabia; 2 King Abdullah International Medical Research Centre (KAIMRC), Riyadh, Saudi Arabia; 3 College of Medicine, King Saud Bin Abdulaziz University for Medical Sciences, Riyadh, Saudi Arabia

**Keywords:** bronchopulmonary dysplasia, neonatal intensive care, non-invasive ventilation, preterm infants, respiratory support

## Abstract

**Aim:**

To evaluate factors associated with death before 36 weeks’ postmenstrual age (PMA) or moderate-to-severe bronchopulmonary dysplasia (BPD) in very preterm infants while accounting for survivor bias.

**Methods:**

This retrospective cohort study included infants born at <32 weeks’ gestation and admitted to a tertiary neonatal intensive care unit between January 2017 and December 2025. The primary outcome was death before 36 weeks’ PMA or moderate-to-severe BPD. Baseline characteristics, neonatal morbidities, and respiratory support variables were examined. Multivariable logistic regression was performed using a sequential modelling approach incorporating early clinical variables and subsequent respiratory-course variables.

**Results:**

Among 1,259 infants, 1,253 had complete outcome data and were included in the analysis; the composite outcome occurred in 27.1%. Risk decreased progressively with advancing gestational age, from 80.2% in infants born at <26 weeks to 11.7% at 30–31 weeks (*p* < 0.001). Each additional week of gestation was associated with lower odds of the composite outcome (aOR: 0.85, 95% CI: 0.77–0.94). In the fully adjusted model, cumulative duration of invasive ventilation (aOR: 1.29 per 7 days, 95% CI: 1.14–1.45) and HFOV exposure (aOR: 1.79, 95% CI: 1.15–2.79) were associated with the composite outcome, although the association with HFOV likely reflected greater underlying respiratory illness severity and escalation of support. Associations involving infection-related variables were attenuated after inclusion of respiratory-course variables. Model discrimination improved from 0.807 to 0.841.

**Conclusions:**

In very preterm infants, lower gestational age and prolonged invasive ventilation were the principal factors associated with death or moderate-to-severe BPD. Associations involving respiratory escalation therapies likely reflected greater underlying illness severity and evolving respiratory trajectory.

## Introduction

Bronchopulmonary dysplasia (BPD) remains one of the most common complications of very preterm birth and is associated with significant long-term respiratory and neurodevelopmental morbidity (1, 2). The condition reflects disrupted lung development in the immature lung following exposure to mechanical ventilation, oxygen therapy, and inflammatory injury during the early neonatal period (3). Despite advances in perinatal and respiratory care, moderate-to-severe forms of BPD continue to affect a substantial proportion of extremely preterm infants (4).

Bronchopulmonary dysplasia was first described by Northway and colleagues in 1967 as a chronic lung disease in preterm infants exposed to prolonged mechanical ventilation and high oxygen concentrations (5). Subsequent definitions evolved toward treatment-based criteria, most notably the requirement for supplemental oxygen for at least 28 days after birth with severity classification at 36 weeks' postmenstrual age, as formalised in the NICHD consensus definition (6). With advances in neonatal respiratory care, including the increasing use of non-invasive ventilation and changes in oxygen targeting, limitations of oxygen-based definitions became apparent, prompting the development of revised classification systems that better reflect contemporary respiratory support practices and improve prognostic accuracy (7, 8). More recently, Jensen and colleagues proposed a respiratory support–based definition that classifies BPD severity according to the level of respiratory support required at 36 weeks' postmenstrual age, independent of oxygen concentration (9). This contemporary framework has been shown to better predict clinically relevant outcomes, including respiratory morbidity, hospital readmission, and neurodevelopmental impairment, and may more accurately capture disease severity than earlier oxygen-based definitions (10–15).

Moderate-to-severe BPD and early mortality represent competing outcomes of extreme prematurity, and analyses restricted to survivors are therefore prone to survivor bias. In addition, several determinants of BPD, including infection and escalation of respiratory support, are closely linked to illness severity, complicating interpretation of treatment outcome associations. Most data describing these relationships are derived from North American, European, or Australasian cohorts, with limited data from the Middle East, where differences in population characteristics and clinical practices may influence outcomes. In this context, we conducted a single-centre cohort study of infants born at <32 weeks' gestation to evaluate a composite outcome of death before 36 weeks' postmenstrual age or moderate-to-severe BPD. We examined associations with perinatal characteristics, neonatal morbidities, and respiratory course variables, and explored whether respiratory trajectory may reflect an intermediate clinical trajectory linking early clinical risk factors to adverse outcomes.

## Methods

### Study design and setting

This retrospective observational cohort study was conducted in the neonatal intensive care unit at King Abdulaziz Medical City, Riyadh, Saudi Arabia, a tertiary referral centre providing level III neonatal intensive care. A consecutive cohort of infants admitted between January 2017 and December 2025 was included.

### Study population

All infants born at <32 weeks' gestation and admitted to the neonatal intensive care unit during the study period were eligible for inclusion. No exclusions were applied based on birth weight, sex, or initial respiratory support modality. Infants who died before 36 weeks' PMA were retained in the analysis and classified within the composite outcome. Infants who were still alive but had not yet reached 36 weeks' postmenstrual age at the time of data extraction could not be classified for BPD and were therefore excluded from outcome-based analyses. The final analytic cohort for outcome analyses comprised 1,253 infants.

### Data sources and data collection

Clinical data were extracted retrospectively from electronic medical records. Data abstraction was performed using standardised templates to ensure consistency across the study period. Extracted variables included perinatal characteristics, respiratory support modalities, cardiovascular interventions, infection-related complications, neurological morbidity, and clinical outcomes recorded during routine care. Data integrity checks were undertaken to ensure consistency of definitions and completeness of outcome ascertainment.

### Exposure and predictor variables

Perinatal variables included gestational age at birth, sex, antenatal corticosteroid exposure, mode of delivery, and premature rupture of membranes. Early neonatal variables included the 5-min Apgar score and surfactant administration, including receipt of two or more doses.

Respiratory variables included initial respiratory support at NICU admission and exposure to high frequency oscillatory ventilation (HFOV) at any time during admission. Infants initially managed with HFOV were included within the HFOV exposure category**.** Respiratory variables also included high flow nasal cannula use, inhaled nitric oxide administration, failed first extubation, and cumulative duration of invasive mechanical ventilation. Failed extubation was defined as reintubation within 72 h following the first planned extubation attempt.

Infection-related variables included late-onset sepsis, defined as positive blood, urine, or cerebrospinal fluid cultures obtained after 72 h of life, and ventilator-associated pneumonia, defined according to CDC/NHSN neonatal criteria as pneumonia occurring after at least 48 h of invasive mechanical ventilation in association with new or progressive radiographic infiltrates, worsening respiratory status, and compatible clinical and microbiological findings (16). Neurological morbidity was defined as severe intraventricular haemorrhage (grade III or IV) (17).

### Outcome definitions

The primary outcome was a composite of death before 36 weeks' PMA or moderate-to -severe BPD.

Death was defined as mortality occurring at any time before 36 weeks' PMA. Among surviving infants, BPD severity was classified at 36 weeks' postmenstrual age according to respiratory support-based criteria proposed by Jensen et al. (9). Moderate-to severe- BPD was defined as Grade 2 or 3 disease, corresponding to the requirement for non-invasive respiratory support, high-flow nasal cannula >2 L/min, or invasive mechanical ventilation at 36 weeks' PMA (9).

Infants discharged before 36 weeks' PMA were clinically stable and off all respiratory support and were therefore classified as having no BPD. Transfer prior to 36 weeks’ PMA was not standard practice in our centre, and outcome data at 36 weeks' PMA were available for almost all infants included in the analytic cohort.

Secondary outcomes included the individual components of the composite outcome, namely death before 36 weeks' PMA and moderate-to-severe BPD among survivors, as well as duration of respiratory support and length of hospital stay. Length of stay was defined as the number of days from birth to discharge or death.

### Handling of competing outcomes

Death before 36 weeks' PMA represents a competing outcome for BPD. To address this, the primary analysis used a composite outcome incorporating both early death and moderate-to-severe BPD, allowing inclusion of all infants and reducing survivor bias.

### Statistical analysis

Baseline characteristics were summarised according to outcome group as survivors with no or mild BPD, survivors with moderate-to-severe BPD, and infants who died before 36 weeks' PMA. Continuous variables are presented as mean with standard deviation or median with interquartile range, as appropriate. Categorical variables are presented as counts and percentages.

Between-group comparisons were performed using one-way analysis of variance for normally distributed variables and Kruskal–Wallis tests for non-normally distributed variables. Categorical variables were compared using chi-square tests. Where overall comparisons were statistically significant, results are presented as overall group differences without formal *post hoc* pairwise testing.

Gestational age–specific gradients in outcomes were examined across predefined gestational age strata using chi-square testing, with assessment of trends across ordered gestational age strata using the Cochran–Armitage trend test.

Associations between clinical variables and the composite outcome of death before 36 weeks' PMA or moderate-to-severe BPD were evaluated using logistic regression. Univariable logistic regression analyses were performed for clinically relevant perinatal characteristics, infection-related variables, neurological morbidity, and respiratory variables associated with the composite outcome.

Multivariable logistic regression modelling was performed using a sequential modelling approach. The first model (Model A) included early clinical and postnatal variables reflecting initial disease burden and complications, including gestational age per week, sex, complete antenatal corticosteroid exposure, receipt of two or more surfactant doses, patent ductus arteriosus treatment, late-onset sepsis, ventilator-associated pneumonia, severe intraventricular haemorrhage, periventricular leukomalacia, pulmonary haemorrhage, and pneumothorax. The second model (Model B) additionally incorporated respiratory-course variables reflecting illness severity and treatment escalation, including exposure to high-frequency oscillatory ventilation, high-flow nasal cannula use, inhaled nitric oxide administration, failed first extubation, postnatal steroid exposure, and cumulative duration of invasive mechanical ventilation. Duration of invasive mechanical ventilation was modelled as a continuous variable scaled per seven-day increase. This sequential modelling approach was designed to evaluate how associations with the outcome evolved following inclusion of variables reflecting increasing clinical complexity and respiratory severity. Several variables included in Model B occurred during the evolving postnatal clinical course and were therefore interpreted as markers of illness severity and respiratory trajectory rather than baseline predictors.

Analyses for both models were performed within the same complete-case dataset to allow direct comparison. No imputation was performed. Adjusted odds ratios with 95% confidence intervals were reported. Model discrimination was assessed using the area under the receiver operating characteristic curve. Calibration was evaluated using the Hosmer–Lemeshow goodness-of-fit test. Improvement in model performance between Model A and Model B was assessed using likelihood ratio testing. Model assumptions, including absence of multicollinearity, were assessed prior to analysis.

Birth weight was not included in multivariable models because of strong collinearity with gestational age, which was retained as the primary maturity-related variable given its closer biological and clinical relationship to pulmonary development and respiratory outcomes in very preterm infants. Although small-for-gestational-age status may provide additional prognostic information, standardised growth centiles and *z*-scores were not consistently available across the full study period using a uniform reference standard, limiting reliable classification across the cohort. In addition, some maternal variables, including hypertensive disorders of pregnancy and chorioamnionitis, as well as neonatal illness severity scores such as CRIB and SNAPPE-II, were not consistently available across the study period and therefore could not be reliably incorporated into adjusted analyses. The absence of these variables introduces the possibility of residual confounding related to baseline illness severity and antenatal inflammatory risk. Model stability was ensured by maintaining an appropriate events-per-variable ratio.

Missing data were assessed for all variables included in regression models. Missingness was low for baseline variables but higher for several postnatal respiratory-course variables that were not consistently applicable to all infants. Multivariable analyses were therefore performed using a complete-case approach. Infants included in the complete-case analyses were compared with those excluded because of incomplete covariate data, and these comparisons are presented in Supplementary Table S1.

All statistical tests were two-sided, and a *p* value of less than 0.05 was considered statistically significant. Analyses were performed using IBM SPSS Statistics version 31.

## Results

A total of 1,259 infants were included in the study cohort. Of these, 1,253 infants had complete outcome data and were included in analyses of the composite endpoint. Infants who had not reached 36 weeks' postmenstrual age at the time of data extraction (*n* = 6) were excluded from outcome-based analyses. Multivariable analyses were performed using a complete-case approach, with 771 infants included in the final regression models.

Table 1 shows baseline characteristics across outcome groups. Analyses are restricted to infants with complete outcome classification (*n* = 1,253). Gestational age decreased progressively from 28.8 ± 2.0 weeks in infants with no or mild bronchopulmonary dysplasia (BPD) to 26.4 ± 2.3 weeks in those with moderate-to-severe BPD and 25.1 ± 2.4 weeks in those who died (*p* < 0.001). Length of stay was longest in infants with moderate-to-severe BPD (109 [90–138] days), whereas infants who died had shorter durations of admission (5.5 [1–14] days; *p* < 0.001). Completion of antenatal corticosteroids and caesarean delivery differed between groups, while sex and premature rupture of membranes were similar.

**Table 1:** Baseline characteristics according to outcome group.

Characteristic	No/Mild BPD (*n* = 914)	Moderate-to-severe BPD (*n* = 199)	Death <36 weeks (*n* = 140)	*p* value
Gestational age, weeks	28.83 ± 2.04	26.35 ± 2.30	25.11 ± 2.36	<0.001
Male sex	501 (54.8%)	109 (54.8%)	80 (57.1%)	0.872
Complete antenatal steroids	574 (62.8%)	124 (62.3%)	68 (48.6%)	0.025
Caesarean delivery	639 (69.9%)	124 (62.3%)	59 (42.1%)	<0.001
PROM	272 (29.8%)	65 (32.7%)	54 (38.6%)	0.078
Apgar score at 5 min	8 [7–9]	7 [6-8]	6.[5–7]	<0.001
Length of stay, days	46 [33–68]	109 [90–138]	5.5 [1–14]	<0.001

Values are mean ± standard deviation (SD), median [interquartile range (IQR)], or *n* (%). Analyses are based on infants with complete outcome data (*n* = 1,253). Infants who had not reached 36 weeks’ postmenstrual age at the time of data extraction (*n* = 6) were excluded from outcome-based analyses. BPD, bronchopulmonary dysplasia; PROM, premature rupture of membranes.

Table 2 demonstrates a clear gestational age–dependent gradient in outcomes. Analyses are based on the full study cohort (*n* = 1,259). The composite outcome decreased from 80.2% in infants born at <26 weeks to 58.7% at 26–27 + 6 weeks, 33.1% at 28–29 + 6 weeks, and 11.7% at 30–31 + 6 weeks (*p* < 0.001 for trend). Early death declined from 36.0% to 1.9%, and moderate-to-severe BPD from 30.4% to 4.7% across the same strata (both *p* < 0.001). Absolute risk reduction increased stepwise with advancing gestational age, reaching −68.5% in infants born at 30–31 + 6 weeks. A strong inverse association between gestational age and adverse outcomes was observed across strata.

**Table 2:** Gradient of early death, moderate-to-severe BPD, and composite outcome by gestational age strata.

Gestational age stratum	Total N	Early death *n* (%)	Moderate-to-severe BPD *n* (%)	Composite outcome *n* (%)	Absolute risk reduction vs <26 weeks
<26 weeks	247	89 (36.0%)	75 (30.4%)	198 (80.2%)	Reference
26–27 + 6 weeks	223	26 (11.7%)	59 (26.5%)	131 (58.7%)	−21.5%
28–29 + 6 weeks	317	16 (5.0%)	43 (13.6%)	105 (33.1%)	−47.1%
30–31 + 6 weeks	472	9 (1.9%)	22 (4.7%)	55 (11.7%)	−68.5%

Values are *n* (%) unless otherwise specified. Analyses are based on the full study cohort (*n* = 1,259). Infants who had not reached 36 weeks’ postmenstrual age at the time of data extraction (*n* = 6) were not eligible for BPD classification**.** Absolute risk reduction represents the difference in composite outcome risk compared with infants born at <26 weeks. The composite outcome is defined as death before 36 weeks’ postmenstrual age or moderate-to-severe bronchopulmonary dysplasia. BPD, bronchopulmonary dysplasia.

Table 3 summarises major comorbidities and interventions across outcome groups. Analyses are restricted to infants with complete outcome classification (*n* = 1,253). Infants with adverse outcomes had higher rates of respiratory interventions, infection-related complications, and severe neurological injury (all *p* < 0.001).

**Table 3:** Major comorbidities and NICU interventions according to outcome group.

Comorbidity/intervention	No/Mild BPD (*n* = 914)	Moderate-to-severe BPD (*n* = 199)	Death <36 weeks (*n* = 140)	*p* value
Respiratory therapies				
Surfactant administered (any)	538 (58.9%)	177 (88.9%)	123 (87.9%)	<0.001
≥2 surfactant doses	227 (24.8%)	103 (51.8%)	89 (63.6%)	<0.001
Cardiovascular interventions				
PDA treatment/closure	96 (10.5%)	69 (34.7%)	9 (6.4%)	<0.001
Infectious complications				
Late-onset sepsis	122 (13.3%)	70 (35.2%)	21 (15.0%)	<0.001
Ventilator-associated pneumonia	40 (4.4%)	61 (30.7%)	8 (5.7%)	<0.001
Neurological injury				
Severe intraventricular hemorrhage (grade III–IV)	60 (6.6%)	43 (21.6%)	39 (27.9%)	<0.001
Periventricular leukomalacia	58 (6.3%)	28 (14.1%)	12 (8.6%)	0.001
Gastrointestinal morbidity				
Surgical necrotizing enterocolitis	21 (2.3%)	35 (17.6%)	10 (7.1%)	<0.001

Analyses are based on infants with complete outcome classification (*n* = 1,253).

Table 4 summarises respiratory support patterns across outcome groups. Analyses are restricted to infants with complete outcome classification (*n* = 1,253). Initial respiratory support differed significantly (*p* < 0.001), with invasive ventilation more common among infants with adverse outcomes. Conventional ventilation was used initially in 65.3% of infants with moderate-to-severe BPD and 57.1% of those who died, compared with 36.9% in infants with no or mild BPD. Initial high-frequency oscillatory ventilation (HFOV) was most frequent among infants who died (36.4%) and uncommon in those with no or mild BPD (3.6%), while non-invasive support was more frequent in infants with favourable outcomes. Escalation therapies showed marked differences between groups. HFOV exposure at any time occurred in 17.0%, 66.3%, and 90.7% of infants, and inhaled nitric oxide use in 7.2%, 44.7%, and 60.7%, respectively (both *p* < 0.001). These variables were strongly associated with adverse outcomes but should be interpreted as markers of evolving illness severity rather than independent predictors. Failed first extubation was more frequent in moderate-to-severe BPD (22.6%) compared with no or mild BPD (4.6%) and death (1.4%) (*p* < 0.001). Duration of respiratory support also differed substantially. Median duration of invasive ventilation was 5 [2–12] days in infants with no or mild BPD and 37 [14–52] days in those with moderate-to-severe BPD, while infants who died had shorter durations (6 [2–13] days) (*p* < 0.001).

**Table 4:** Respiratory support course and therapies according to outcome group.

Respiratory variable	No/Mild BPD (*n* = 914)	Moderate-to-severe BPD (*n* = 199)	Death <36 weeks (*n* = 140)	*p* value
Initial respiratory support				<0.001
CPAP	219 (24.0%)	12 (6.0%)	1 (0.7%)	
NIMV	320 (35.0%)	30 (15.1%)	6 (4.3%)	
Intubated—conventional ventilation	337 (36.9%)	130 (65.3%)	80 (57.1%)	
Intubated—HFOV	33 (3.6%)	26 (13.1%)	51 (36.4%)	
Other (NC)	5 (0.5%)	1 (0.5%)	1 (0.7%)	
Respiratory escalation therapies				
HFOV required at any time	155 (17.0%)	132 (66.3%)	127 (90.7%)	<0.001
HFNC required at any time	535 (58.5%)	165 (82.9%)	5 (3.6%)	<0.001
Inhaled nitric oxide	66 (7.2%)	89 (44.7%)	85 (60.7%)	<0.001
Failed first extubation	42 (4.6%)	45 (22.6%)	2 (1.4%)	<0.001
Any postnatal steroids	163 (17.8%)	128 (64.3%)	33 (23.6%)	<0.001
Duration of respiratory support				
Days intubated	5 [2–12]	37 [14–52]	6 [2–13]	<0.001
Days on non-invasive support†	5 [2–13]	25.5 [12–40]	7.5 [2.3–18.5]	<0.001
HFNC days	7 [4–13.8]	17 [9–27]	7 [6–10]	<0.001
Duration of iNO, days	4 [3–9.8]	15 [6–24]	5 [2–9]	<0.001

Values are *n* (%) or median [IQR]. Analyses are based on infants with complete outcome classification (*n* = 1,253). CPAP, continuous positive airway pressure; NIMV, nasal intermittent mandatory ventilation; HFOV, high-frequency oscillatory ventilation; HFNC, high-flow nasal cannula; NC, nasal cannula; iNO, inhaled nitric oxide.

Table 5 presents multivariable associations with the composite outcome using a sequential modelling approach. Analyses were restricted to infants with complete outcome classification (*n* = 1,253), with multivariable analyses conducted on the complete-case dataset (*n* = 771). In Model A, lower gestational age and markers of infection and lung injury were associated with higher odds of the composite outcome. After inclusion of respiratory-course variables in Model B, gestational age remained associated with outcome, while longer duration of invasive ventilation showed the strongest association. Associations involving respiratory-course variables likely reflect illness severity given their temporal overlap with outcome development. HFOV exposure was also associated with higher odds of the composite outcome. Model discrimination improved with inclusion of respiratory-course variables (AUC: 0.807–0.841).

**Table 5:** Multivariable associations with the composite endpoint.

Predictor	Model A aOR (95% CI)	*p* value	Model B aOR (95% CI)	*p* value
Gestational age, per 1 week increase	0.73 (0.67–0.79)	<0.001	0.85 (0.77–0.94)	0.001
Male sex	1.26 (0.90–1.75)	0.177	1.21 (0.85–1.72)	0.284
Complete antenatal steroids	1.18 (0.84–1.66)	0.353	1.12 (0.78–1.62)	0.527
≥2 surfactant doses	1.53 (1.09–2.13)	0.013	1.18 (0.82–1.71)	0.364
PDA treatment/closure	1.49 (0.96–2.30)	0.074	1.14 (0.71–1.82)	0.597
Late-onset sepsis	2.11 (1.38–3.22)	<0.001	1.56 (1.00–2.45)	0.052
Ventilator-associated pneumonia	3.60 (1.94–6.66)	<0.001	1.52 (0.76–3.05)	0.240
Severe IVH (grade III–IV)	1.64 (0.98–2.76)	0.062	1.16 (0.65–2.06)	0.614
PVL	1.34 (0.75–2.39)	0.322	1.20 (0.65–2.23)	0.554
Pulmonary haemorrhage	2.59 (1.05–6.38)	0.039	1.95 (0.77–4.89)	0.157
Pneumothorax	1.04 (0.39–2.80)	0.930	0.75 (0.28–2.04)	0.575
HFOV exposure	—	—	1.79 (1.15–2.79)	0.010
HFNC exposure	—	—	0.74 (0.49–1.09)	0.129
Inhaled nitric oxide	—	—	1.32 (0.76–2.27)	0.325
Failed first extubation	—	—	1.42 (0.78–2.57)	0.252
Postnatal steroids	—	—	1.27 (0.84–1.92)	0.264
Duration of invasive ventilation (per 7 days)	—	—	1.29 (1.14–1.45)	<0.001

Adjusted odds ratios were estimated using multivariable logistic regression. Analyses are based on infants with complete outcome classification (*n* = 1,253). Multivariable models were based on 771 infants with complete data. Model A includes early clinical and postnatal variables, and Model B additionally incorporates respiratory-course variables. Respiratory-course variables may overlap temporally with outcome development and should be interpreted as markers of illness severity rather than causal predictors**.** AUC increased from 0.807 (Model A) to 0.841 (Model B).

Table 6 presents univariable associations with the composite outcome. Analyses were restricted to infants with complete outcome classification (*n* = 1,253). Lower gestational age and multiple markers of respiratory severity, infection-related complications, and neurological injury were associated with increased odds of the composite outcome. These associations should be interpreted cautiously, as several variables reflect the evolving clinical course and may overlap temporally with outcome development, and are therefore subject to potential reverse causation and confounding by indication.

**Table 6:** Univariable associations with the composite endpoint (death before 36 weeks or moderate-to-severe BPD).

Predictor	Unadjusted OR (95% CI)	*p* value
Gestational age (per week)	0.57 (0.54–0.61)	<0.001
Complete antenatal steroids	1.06 (0.87–1.28)	0.568
≥2 surfactant doses	3.90 (3.05–4.99)	<0.001
PDA treatment	1.38 (1.23–1.54)	<0.001
HFOV (any exposure)	12.46 (9.33–16.63)	<0.001
HFNC (any exposure)	0.73 (0.58–0.92)	0.008
Inhaled nitric oxide	11.19 (7.86–15.95)	<0.001
Failed first extubation	3.54 (2.20–5.69)	<0.001
Ventilator-associated pneumonia	11.46 (6.65–19.75)	<0.001
Severe IVH (grade III–IV)	5.16 (3.51–7.59)	<0.001
PVL	2.50 (1.65–3.80)	<0.001
Postnatal steroids	2.44 (2.09–2.85)	<0.001

Odds ratios represent univariable logistic regression estimates. Analyses are based on infants with complete outcome classification (*n* = 1,253). Variables occurring during the postnatal course (e.g., HFOV exposure, postnatal steroids, duration of ventilation) should be interpreted as markers of evolving illness severity rather than causal predictors.

## Discussion

In this large single-centre cohort of very preterm infants, we evaluated determinants of a composite outcome incorporating death before 36 weeks' postmenstrual age and moderate-to-severe bronchopulmonary dysplasia, thereby addressing survivor bias inherent in analyses restricted to survivors. Outcomes demonstrated a clear gradient according to gestational maturity, with lower gestational age associated with higher risk. In multivariable analyses, gestational age and cumulative duration of invasive mechanical ventilation were the principal factors associated with the composite outcome. Associations with infection-related complications and neurological injury were attenuated after adjustment for respiratory-course variables, suggesting that respiratory trajectory may reflect the evolution of illness severity linking early clinical risk factors to adverse outcomes, rather than representing a causal mediating pathway. By incorporating early mortality into the outcome definition, this approach captures a broader spectrum of clinically relevant outcomes. These findings are consistent with a pathway linking early clinical risk factors to adverse outcomes, with respiratory trajectory reflecting evolving disease severity rather than acting as a direct causal mechanism.

Outcome patterns across gestational strata demonstrated a clear inverse association between gestational age and adverse outcomes, with stepwise reductions in the composite endpoint as maturity increased. This finding aligns with established evidence linking prematurity to pulmonary morbidity. Infants born at earlier gestational ages have structurally and functionally immature lungs, including reduced alveolarisation, impaired surfactant production, and underdeveloped pulmonary vasculature, which increase susceptibility to ventilator-induced lung injury and inflammatory pathways implicated in the development of bronchopulmonary dysplasia. Large cohort studies and systematic reviews have consistently identified gestational age as the strongest predictor of both BPD and early mortality in very preterm infants (18–20). These findings support the central role of biological maturity in determining respiratory outcomes during the neonatal period and are consistent with an association between gestational age and subsequent respiratory trajectory.

In addition to gestational maturity, infection-related and inflammatory morbidities were associated with adverse outcomes. Late-onset sepsis and ventilator-associated pneumonia were more frequent among infants who developed moderate-to-severe BPD, consistent with evidence linking systemic and pulmonary inflammation to the pathogenesis of chronic lung disease. Infection may exacerbate lung injury through cytokine-mediated inflammatory responses, oxidative stress, and disruption of alveolar and vascular development. Previous studies have demonstrated associations between late-onset sepsis, nosocomial infection, and increased risk of severe BPD or death (21–23). In the present study, these associations were attenuated after adjustment for respiratory-course variables, suggesting that infection-related morbidity is associated with adverse outcomes in the context of greater illness severity and increased need for prolonged invasive respiratory support, rather than representing a direct causal pathway.

Building on these findings, respiratory course variables, particularly cumulative duration of invasive mechanical ventilation, showed the strongest associations with outcome, suggesting that respiratory trajectory may reflect the cumulative burden of illness severity arising from multiple upstream risk factors rather than representing a direct causal pathway. Infants who developed moderate-to-severe bronchopulmonary dysplasia required longer periods of invasive ventilation compared with those with no or mild disease. This finding is consistent with the established association between ventilator exposure and the development of chronic lung disease in preterm infants. Prolonged mechanical ventilation is known to be associated with lung injury through mechanisms such as barotrauma, volutrauma, and biotrauma, leading to inflammatory responses that disrupt alveolar and vascular development. Prior studies have similarly identified duration of invasive ventilation as a key factor associated with moderate-to-severe bronchopulmonary dysplasia and respiratory morbidity (9, 24, 25). More recent analyses have also shown that early respiratory instability and prolonged exposure to invasive ventilation are associated with subsequent chronic lung disease, independent of gestational age and birth weight (14). These findings are consistent with current clinical approaches that aim to minimise exposure to invasive ventilation; however, such associations should be interpreted cautiously in observational analyses, as they may reflect underlying illness severity rather than direct causal effects.

In our study, several therapies associated with adverse outcomes in unadjusted analyses did not remain significantly associated with the composite outcome after adjustment for respiratory severity. Exposure to inhaled nitric oxide was more frequent among infants with adverse outcomes but did not retain a statistically significant association in multivariable models. This pattern is consistent with confounding by indication, whereby therapies are preferentially administered to infants with more severe respiratory failure rather than indicating a direct treatment effect. These findings align with evidence from randomised controlled trials and meta-analyses, which have shown that inhaled nitric oxide does not reduce the risk of bronchopulmonary dysplasia or death in very preterm infants when used routinely (26–29). Recent guideline statements and expert consensus recommendations do not support routine use of inhaled nitric oxide for prevention of bronchopulmonary dysplasia in preterm infants and restrict its use to selected cases with documented pulmonary hypertension (30, 31). Overall, these observations are consistent with the interpretation that the association between inhaled nitric oxide exposure and adverse outcomes may reflect underlying disease severity rather than a direct treatment effect, highlighting the importance of accounting for respiratory trajectory when interpreting associations between therapies and clinical outcomes in observational studies.

Findings from large neonatal network cohorts further support the importance of respiratory trajectory in association with pulmonary outcomes. Multicentre analyses have consistently shown that prolonged invasive mechanical ventilation is strongly associated with the development of moderate-to-severe BPD, independent of gestational age and birth weight (21, 22). In addition, early respiratory instability and escalation of respiratory support have been linked to subsequent BPD, highlighting the association between respiratory trajectory and adverse outcomes (32, 33). In our study, exposure to HFOV remained associated with the composite outcome after adjustment, likely reflecting greater underlying respiratory severity and escalation of ventilatory support rather than a direct treatment effect. Randomised trials and meta-analyses have shown inconsistent effects of HFOV on BPD and mortality, with no clear overall benefit when used routinely (34). More recent evidence suggests that HFOV may reduce the incidence of BPD in selected populations of preterm infants, although uncertainty regarding optimal patient selection persists (35).

In addition to these mechanistic and clinical considerations, regional data provide important context for interpreting these findings. Data from the Gulf region remain limited but demonstrate similar associations between respiratory severity, prolonged ventilation, and adverse pulmonary outcomes in preterm infants. A large level III neonatal intensive care cohort from Qatar including infants ≤32 weeks' gestation reported a bronchopulmonary dysplasia incidence of approximately 29%, with ductal pathology and clinical factors identified as key predictors (36). Similarly, a cohort from Oman reported a combined outcome of bronchopulmonary dysplasia or death in 28.4% of infants, with duration of ventilation and intraventricular haemorrhage among the strongest associated factors (37). Earlier data from Kuwait also demonstrated associations between prolonged ventilation and the development of bronchopulmonary dysplasia (38). Within this context, the present cohort adds to the limited regional literature by evaluating bronchopulmonary dysplasia using current severity definitions and incorporating early mortality within the outcome framework, thereby reducing survivor bias and allowing a broader assessment of clinically relevant respiratory outcomes.

This study has several strengths. It includes a large cohort of very preterm infants managed within a single tertiary neonatal intensive care unit, providing relative consistency in clinical practices and enabling detailed evaluation of respiratory trajectories and associated outcomes. The inclusion of both early mortality and bronchopulmonary dysplasia within a composite endpoint reduces survivor bias and allows a more complete assessment of adverse respiratory outcomes. In addition, the integration of early perinatal characteristics with longitudinal respiratory course variables provides a comprehensive evaluation of variables associated with adverse outcomes. The study also contributes data from an underrepresented Middle Eastern population, supporting regional benchmarking and contextual interpretation of outcomes.

Several limitations should be considered. The retrospective single-centre design limits causal inference and introduces the possibility of residual confounding despite multivariable adjustment. However, the analysis incorporated a broad range of perinatal, respiratory, and neonatal morbidity variables using sequential multivariable models, with assessment of collinearity and model stability to strengthen the robustness of the findings. Clinical practices, including respiratory support strategies and use of non-invasive ventilation, evolved over the study period and may have influenced the observed associations. To address this, analyses were performed across predefined clinical eras and adjusted models incorporated key baseline perinatal variables. Temporal relationships between respiratory interventions and outcomes also cannot be fully established for variables that evolved during the clinical course. The absence of maternal inflammatory variables, including chorioamnionitis, and neonatal illness severity scores such as CRIB and SNAPPE-II, which were not consistently available across the study period, may have contributed to incomplete adjustment for baseline illness severity and antenatal inflammatory risk. Nevertheless, several clinically relevant proxies of illness severity, including gestational age, respiratory support requirements, invasive ventilation exposure, major neonatal morbidities, and escalation therapies, were included in the adjusted analyses. Multivariable analyses were performed using a complete-case approach because several respiratory-course variables were not consistently applicable across the cohort. Although comparisons between included and excluded infants were performed to assess potential selection bias, residual bias cannot be fully excluded. The proportion of missing baseline data was low, and standardized institutional electronic records helped support data consistency across the study period. Finally, although small-for-gestational-age status may provide additional prognostic information, standardised growth centiles and *z*-scores were not consistently available across the full study period using a uniform reference standard, limiting reliable classification across the cohort. Despite these limitations, the large cohort size, consistent institutional practices, and coherence of the observed findings support the overall validity of the study.

## Conclusion

In this large single-centre cohort of very preterm infants, gestational maturity and cumulative duration of invasive mechanical ventilation were the principal factors associated with death before 36 weeks' postmenstrual age or moderate-to-severe bronchopulmonary dysplasia. Associations with infection-related complications and neurological injury were attenuated after adjustment for respiratory course variables, suggesting that respiratory trajectory may reflect the evolution of illness severity and is consistent with a pathway linking early clinical risk factors to adverse outcomes. These findings are consistent with a framework in which respiratory trajectory reflects the integration of multiple risk factors associated with adverse outcomes, highlighting the potential relevance of clinical strategies aimed at minimising exposure to invasive ventilation within the context of overall illness severity.

## Data Availability

The raw data supporting the conclusions of this article will be made available by the authors, without undue reservation.
