# Supplementary material for: Respiratory trajectory and risk of death or moderate-to-severe bronchopulmonary dysplasia in very preterm infants: a nine-year cohort study
Source: Front Pediatr. 2026 Jun 1;14:1843270. doi: 10.3389/fped.2026.1843270 (PMC13265491; doi:10.3389/fped.2026.1843270)
Supplement: Supplementary file 1 [file Table1.docx]

**Supplementary Table S1. Baseline characteristics of infants included and excluded from the complete-case multivariable analysis**

| **Characteristic** | **Included in complete-case models (n=771)** | **Excluded from complete-case models (n=482)** | **p value** |
| --- | --- | --- | --- |
| Gestational age, weeks | 27.5 ± 2.4 | 28.9 ± 2.5 | <0.001 |
| Birth weight, g | 1062 ± 362 | 1271 ± 409 | <0.001 |
| Male sex | 423 (54.8%) | 267 (55.4%) | 0.839 |
| Complete antenatal steroids | 480 (62.2%) | 300 (62.2%) | 0.985 |
| Caesarean delivery | 532 (69.0%) | 287 (59.6%) | <0.001 |
| PROM | 236 (30.6%) | 159 (33.0%) | 0.385 |
| 5-minute Apgar score | 8 [7–8] | 8 [7–9] | <0.001 |
| Composite outcome | 349 (45.3%) | 135 (28.1%) | <0.001 |
| Death before 36 weeks PMA | 42 (5.5%) | 101 (20.9%) | <0.001 |
